# Supplementary material for: Neutrophil count multiplied by D-dimer combined with pneumonia may better predict short-term outcomes in patients with acute ischemic stroke
Source: PLoS One. 2022 Oct 7;17(10):e0275350. doi: 10.1371/journal.pone.0275350 (PMC9543623; doi:10.1371/journal.pone.0275350)
Supplement: S1 Checklist — (DOCX) [file pone.0275350.s001.docx]

STROBE Statement—checklist of items that should be included in reports of observational studies

|  | | | Item No. | Recommendation | Page  No. | | | Relevant text from manuscript |
| --- | --- | --- | --- | --- | --- | --- | --- | --- |
| **Title and abstract** | | | 1 | (*a*) Indicate the study’s design with a commonly used term in the title or the abstract | 1-2 | | | **Title:** Neutrophil count multiplied by D-dimer combined with pneumonia may better predict short-term outcomes in patients with acute ischemic stroke  **Abstract** |
|  |  |  |  | (*b*) Provide in the abstract an informative and balanced summary of what was done and what was found | 2 | | | **Abstract** |
| Introduction | | | | | | | |  |
| Background/rationale | | | 2 | Explain the scientific background and rationale for the investigation being reported | 3 | | | **Introduction** |
| Objectives | | | 3 | State specific objectives, including any prespecified hypotheses | 3 | | | In order to identify more practical predictors in the laboratory and provide tools for AIS clinical diagnosis and treatment |
| Methods | | | | | | | |  |
| Study design | | | 4 | Present key elements of study design early in the paper | 3-4 | | | This study recorded the clinical manifestations, medical history and a series of laboratory items of AIS patients during hospitalization through follow-up analysis. |
| Setting | | | 5 | Describe the setting, locations, and relevant dates, including periods of recruitment, exposure, follow-up, and data collection | 3-4 | | | Ischemic encephalopathy patients in the First Affiliated Hospital of Harbin Medical University were the focus during July 2019 to July 2021.  All patients were followed up for 1 month to evaluate the short-term prognosis.  Clinical information and laboratory item data collection |
| Participants | | | 6 | (*a*) *Cohort study*—Give the eligibility criteria, and the sources and methods of selection of participants. Describe methods of follow-up  *Case-control study*—Give the eligibility criteria, and the sources and methods of case ascertainment and control selection. Give the rationale for the choice of cases and controls  *Cross-sectional study*—Give the eligibility criteria, and the sources and methods of selection of participants | 4 | | | *Cross-sectional study-* *AIS was diagnosed according to the Chinese Guidelines for the Diagnosis and Treatment of Acute Ischemic Stroke 2018. The patients were all elder than 18 years old* |
|  |  |  |  | (*b*) *Cohort study*—For matched studies, give matching criteria and number of exposed and unexposed  *Case-control study*—For matched studies, give matching criteria and the number of controls per case |  | | |  |
| Variables | | | 7 | Clearly define all outcomes, exposures, predictors, potential confounders, and effect modifiers. Give diagnostic criteria, if applicable | 4 | | | All patients were followed up for 1 month to chase the short-term poor outcome (defined as poor state, drowsiness, lethargy, coma, death, a minimum of just 1 of these criteria should be flagged as having a poor outcome) recorded in the medical history. |
| Data sources/ measurement | | | 8* | For each variable of interest, give sources of data and details of methods of assessment (measurement). Describe comparability of assessment methods if there is more than one group | *4-5* | | | Laboratory data collection |
| Bias | | | 9 | Describe any efforts to address potential sources of bias | 4 | | | Those whose clinical information or laboratory items data were incomplete would be excluded. |
| Study size | | | 10 | Explain how the study size was arrived at | 5 | | | Sample size estimation. |
| Quantitative variables | | 11 | | Explain how quantitative variables were handled in the analyses. If applicable, describe which groupings were chosen and why | 4 | | Clinical information & Laboratory data collection | |
| Statistical methods | | 12 | | (*a*) Describe all statistical methods, including those used to control for confounding | 6 | | Statistical analysis | |
|  |  |  |  | (*b*) Describe any methods used to examine subgroups and interactions | 6 | | Statistical analysis | |
|  |  |  |  | (*c*) Explain how missing data were addressed | 6 | | Statistical analysis | |
|  |  |  |  | (*d*) *Cohort study*—If applicable, explain how loss to follow-up was addressed  *Case-control study*—If applicable, explain how matching of cases and controls was addressed  *Cross-sectional study*—If applicable, describe analytical methods taking account of sampling strategy | 6 | | Statistical analysis | |
|  |  |  |  | (*e*) Describe any sensitivity analyses | 6 | | Statistical analysis | |
| Results | | | | | | | | |
| Participants | | 13* | | (a) Report numbers of individuals at each stage of study—eg numbers potentially eligible, examined for eligibility, confirmed eligible, included in the study, completing follow-up, and analysed | 7 | | There were 1856 AIS patients in total (shown in Fig. 1), 120 (6.47%) patients had poor outcomes. | |
|  |  |  |  | (b) Give reasons for non-participation at each stage | 7 | | Fig 1 | |
|  |  |  |  | (c) Consider use of a flow diagram | 7 | | Fig 1 | |
| Descriptive data | | 14* | | (a) Give characteristics of study participants (eg demographic, clinical, social) and information on exposures and potential confounders | 7 | | Many of the patients had co-existing clinical problems such as…… (Fig 2). | |
|  |  |  |  | (b) Indicate number of participants with missing data for each variable of interest | 7 | | Fig 1 | |
|  |  |  |  | (c) *Cohort study*—Summarise follow-up time (eg, average and total amount) | N/A | |  | |
| Outcome data | | 15* | | *Cohort study*—Report numbers of outcome events or summary measures over time | N/A | |  | |
|  |  |  |  | *Case-control study—*Report numbers in each exposure category, or summary measures of exposure | N/A | |  | |
|  |  |  |  | *Cross-sectional study—*Report numbers of outcome events or summary measures | *7* | | *120 (6.47%) patients had poor outcomes* | |
| Main results | | 16 | | (*a*) Give unadjusted estimates and, if applicable, confounder-adjusted estimates and their precision (eg, 95% confidence interval). Make clear which confounders were adjusted for and why they were included | 10-11 | | Neutrophil count multiplied by D-dimer (NDM) had good regression relationship with poor outcomes of AIS patients. | |
|  |  |  |  | (*b*) Report category boundaries when continuous variables were categorized | 12 | | Neutrophil count multiplied by D-dimer (NDM) had good regression relationship with poor outcomes of AIS patients. | |
|  |  |  |  | (*c*) If relevant, consider translating estimates of relative risk into absolute risk for a meaningful time period | 13 | | NDM (quintile) combined with pneumonia may better predict short-term outcomes in patients with acute ischemic stroke | |
| Other analyses | 17 | | Report other analyses done—eg analyses of subgroups and interactions, and sensitivity analyses | | 9-10 | The 5 quintiles result could be used to analyse the regression and draw the ROC curve. | | |
| Discussion | | | | | | | | |
| Key results | 18 | | Summarise key results with reference to study objectives | | 14 | NDM, short for neutrophil count multiplied by D-dimer, was a new set of data relationships in this study | | |
| Limitations | 19 | | Discuss limitations of the study, taking into account sources of potential bias or imprecision. Discuss both direction and magnitude of any potential bias | | 17-18 | There are some limitations of this study. | | |
| Interpretation | 20 | | Give a cautious overall interpretation of results considering objectives, limitations, multiplicity of analyses, results from similar studies, and other relevant evidence | | 18 | Conclusions | | |
| Generalisability | 21 | | Discuss the generalisability (external validity) of the study results | | 18 | Conclusions | | |
| Other information | | |  | | | | | |
| Funding | 22 | | Give the source of funding and the role of the funders for the present study and, if applicable, for the original study on which the present article is based | | 19 | Funding | | |

*Give information separately for cases and controls in case-control studies and, if applicable, for exposed and unexposed groups in cohort and cross-sectional studies.

**Note:** An Explanation and Elaboration article discusses each checklist item and gives methodological background and published examples of transparent reporting. The STROBE checklist is best used in conjunction with this article (freely available on the Web sites of PLoS Medicine at http://www.plosmedicine.org/, Annals of Internal Medicine at http://www.annals.org/, and Epidemiology at http://www.epidem.com/). Information on the STROBE Initiative is available at www.strobe-statement.org.
