# Supplementary material for: Neutrophil count multiplied by D-dimer combined with pneumonia may better predict short-term outcomes in patients with acute ischemic stroke
Source: PLoS One. 2022 Oct 7;17(10):e0275350. doi: 10.1371/journal.pone.0275350 (PMC9543623; doi:10.1371/journal.pone.0275350)
Supplement: S1 Table — (DOCX) [file pone.0275350.s002.docx]

**S1 Table: The detection materials and methods for laboratory subitems.**

| Items | Subitems abbreviations | Full Name of Subitems | Unit | Methodology | Testing instrument | Sample | Pre-processing mode |
| --- | --- | --- | --- | --- | --- | --- | --- |
| Blood routine test | NEU# | Absolute neutrophil count | *10e+6/L | Flow cytometry | Sysmex XN1800 | Fresh peripheral venous blood sample.  The acquisition tube is a 2.0 mg/mL EDTA-K_2_ anticoagulant venous collection tube (3mL). | The samples were fully mixed and tested on the test instrument. |
|  | LYMPH# | Absolute lymphocyte count | *10e+6/L | Flow cytometry |  |  |  |
|  | MONO# | Absolute monocyte count | *10e+6/L | Flow cytometry |  |  |  |
|  | ESO# | Absolute eosinophil count | *10e+6/L | Flow cytometry |  |  |  |
|  | BASO# | Absolute basophil count | *10e+6/L | Flow cytometry |  |  |  |
|  | HGB | Hemoglobin | g/L | SDS |  |  |  |
|  | RDW | Red blood cell distribution width | % | Flow cytometry |  |  |  |
|  | PLT | Platelet | *10e+6/L | Flow cytometry |  |  |  |
|  | PDW | Platelet distribution width | % | Flow cytometry |  |  |  |
| Coagulation and fibrinolysis test | PT | Prothrombin time | sec | Thrombin time | Sekisui CP3000 | Fresh peripheral venous blood sample.  The acquisition tube is a 109 mmol/L sodium citrate anticoagulant venous collection tube (3mL). | After the sample was collected, the sample was centrifuged for 3500 rpm,10 min, and then tested on the instrument. |
|  | PTA | Prothrombin activity | % | Thrombin time |  |  |  |
|  | PTINR | International Normalized Ratio | INR | Thrombin time |  |  |  |
|  | APTT | Activated Partial Thromboplastin Time | sec | Thrombin time |  |  |  |
|  | FIB | Fibrinogen | g/L | Thrombin time |  |  |  |
|  | D-DIMER | D-dimer | μg/mL, (ddu) | Immunoturbidimetry |  |  |  |
| Chemical and immunology test | HCY | Homocysteine | μmol/L | Enzyme circulation | Abbott Architect C16000+I2000 | Fresh peripheral venous blood sample.  The acquisition tube is biochemical tube, containing coagulant (3mL). | After 30 min of sample collection, the samples were centrifuged at 3500 rpm for 10 min and then tested on the instrument. |
|  | ALB | Albumin | g/L | Biuret |  |  |  |
|  | PAB | Proalbumin | mg/L | Immunoturbidimetry |  |  |  |
|  | GLU | Glucose | mmol/L | Hexokinase |  |  |  |
|  | CHOL | Cholesterol | mmol/L | Cholesterol oxidase |  |  |  |
|  | TG | Triglyceride | mmol/L | GPO - PAP |  |  |  |
|  | HDL-C | High density lipoprotein cholesterol | mmol/L | Reaction promoter peroxidase scavenging |  |  |  |
|  | LDL-C | Low density lipoprotein cholesterol | mmol/L | Surfactant removal |  |  |  |
|  | LDL/HDL ratio | The ratio of LDL to HDL | % | Calculate |  |  |  |
|  | APOA | Apolipoprotein A | g/L | Immunoturbidimetry |  |  |  |
|  | APOB | Apolipoprotein B | g/L | Immunoturbidimetry |  |  |  |
|  | APOA/APOB ratio | The ratio of APOA to APOB | % | Calculate |  |  |  |
|  | Lpa | Lipoprotein a | mg/L | Immunoturbidimetry |  |  |  |
|  | FOLATE | Folate acid | ng/mL | Chemiluminescence |  |  |  |
|  | B12 | Vitamin B12 | pg/mL | Chemiluminescence |  |  |  |
