# Supplementary material for: Neutrophil count multiplied by D-dimer combined with pneumonia may better predict short-term outcomes in patients with acute ischemic stroke
Source: PLoS One. 2022 Oct 7;17(10):e0275350. doi: 10.1371/journal.pone.0275350 (PMC9543623; doi:10.1371/journal.pone.0275350)
Supplement: S2 Table — This table only shows the items that are significantly correlated with the poor prognosis of AIS. The items were grouped into 5 quintiles as grade variables (Q1-Q5). NEU#: Absolute neutrophil count, LYMPH#: Absolute lymphocyte count, MONO#: Absolute monocyte count, ESO#: Absolute eosinophil count, RDW: Red blood cell distribution width, PLT: Platelet, PDW: Platelet distribution width, PT: Prothrombin time, PTA: Prothrombin activity, PTINR: International Normalized Ratio, FIB: Fibrinogen, D-DIMER: D-dimer, HCY: Homocysteine, ALB: Albumin, PAB: Proalbumin, TG: Triglyceride, APOA/APOB ratio: The ratio of APOA to APOB, Lpa: Lipoprotein a. P value1 indicates for the difference result by Mann-Whitney Test, P value2 indicates for the correlated relationship by Spearman’s rho Nonparametric Correlations. (DOCX) [file pone.0275350.s003.docx]

**S2 Table Details of difference and correlated relationship analysis in quintile form of significant continuous measurement data between Group P with Group W.**

| Subitems | Q1 | Q2 | Q3 | Q4 | Q5 | *P* value^1^ | *P* value^2^ |
| --- | --- | --- | --- | --- | --- | --- | --- |
| NEU# (quintile) | <= 3.42 | 3.43 - 4.19 | 4.20 - 5.16 | 5.17 - 6.66 | 6.67+ | <0.001 | <0.001 |
| LYMPH# (quintile) | <= 1.11 | 1.12 - 1.49 | 1.50 - 1.78 | 1.79 - 2.21 | 2.22+ | <0.001 | <0.001 |
| MONO# (quintile) | <= 0.34 | 0.35 - 0.42 | 0.43 -0.51 | 0.52 - 0.64 | 0.65+ | <0.001 | <0.001 |
| ESO# (quintile) | <= 0.03 | 0.04 - 0.07 | 0.08 - 0.11 | 0.12 - 0.19 | 0.20+ | <0.001 | <0.001 |
| RDW (quintile) | <= 37.10 | 37.11 - 40.50 | 40.51 - 42.60 | 42.61 - 45.20 | 45.21+ | <0.001 | <0.001 |
| PLT (quintile) | <= 167.00 | 167.01 - 201.00 | 201.01 - 231.00 | 231.01 - 269.00 | 269.01+ | 0.048 | 0.048 |
| PDW (quintile) | <= 10.90 | 10.91 - 12.20 | 12.21 - 13.50 | 13.51 - 15.90 | 15.91+ | 0.029 | 0.028 |
| PT (quintile) | <= 11.20 | 11.21 - 11.70 | 11.71 - 12.20 | 12.21 - 12.90 | 12.91+ | <0.001 | <0.001 |
| PTA (quintile) | <= 73.40 | 73.41 - 89.00 | 89.01 - 98.30 | 98.31 - 108.60 | 108.61+ | <0.001 | <0.001 |
| PTINR (quintile) | <= 0.96 | 0.97 - 1.01 | 1.02 - 1.06 | 1.07 - 1.12 | 1.13+ | <0.001 | <0.001 |
| FIB (quintile) | <= 2.25 | 2.26 - 2.75 | 2.76 - 3.24 | 3.25 - 3.94 | 3.95+ | 0.004 | 0.004 |
| D-dimer (quintile) | <= 0.48 | 0.49 - 0.64 | 0.65 - 1.14 | 1.15 - 3.09 | 3.10+ | <0.001 | <0.001 |
| HCY (quintile) | <= 9.70 | 9.71 - 12.10 | 12.11 - 14.75 | 14.76 - 20.93 | 20.94+ | <0.001 | <0.001 |
| ALB (quintile) | <= 34.60 | 34.61 - 36.90 | 36.91 - 38.70 | 38.71 - 40.61 | 40.62+ | 0.036 | 0.036 |
| PAB (quintile) | <= 176.00 | 176.01 - 205.00 | 205.01 - 232.00 | 232.01 - 267.05 | 267.06+ | <0.001 | <0.001 |
| TG (quintile) | <= 0.93 | 0.94 - 1.23 | 1.24 - 1.58 | 1.59 - 2.16 | 2.17+ | 0.008 | 0.008 |
| APOA/APOB ratio (quintile) | <= 0.93 | 0.94 - 1.11 | 1.12 - 1.30 | 1.31 - 1.61 | 1.62+ | 0.012 | 0.012 |
| Lpa (quintile) | <= 11.20 | 11.21 - 32.50 | 32.51 - 92.67 | 92.68 - 236.87 | 236.88+ | <0.001 | <0.001 |
| Age (quintile) | <= 57.00 | 57.01 - 64.00 | 64.01 - 69.00 | 69.01 - 77.00 | 77.01+ | <0.001 | <0.001 |

Note: This table only shows the items that are significantly correlated with the poor prognosis of AIS. The items were grouped into 5 quintiles as grade variables (Q1-Q5). NEU#: Absolute neutrophil count, LYMPH#: Absolute lymphocyte count, MONO# : Absolute monocyte count, ESO#: Absolute eosinophil count, RDW: Red blood cell distribution width, PLT: Platelet, PDW: Platelet distribution width, PT: Prothrombin time, PTA: Prothrombin activity, PTINR: International Normalized Ratio, FIB: Fibrinogen, D-DIMER: D-dimer, HCY: Homocysteine, ALB: Albumin, PAB: Proalbumin, TG: Triglyceride, APOA/APOB ratio: The ratio of APOA to APOB, Lpa: Lipoprotein a. P value^1^ indicates for the difference result by Mann-Whitney Test, P value^2^ indicates for the correlated relationship by Spearman's rho Nonparametric Correlations.
