# Supplementary material for: Neutrophil count multiplied by D-dimer combined with pneumonia may better predict short-term outcomes in patients with acute ischemic stroke
Source: PLoS One. 2022 Oct 7;17(10):e0275350. doi: 10.1371/journal.pone.0275350 (PMC9543623; doi:10.1371/journal.pone.0275350)
Supplement: S3 Table — A: Enter method to check single item. B: Only significant laboratory items were included for forward likelihood ratio detection. C: Only clinically significant items were included for forward likelihood ratio test. “/” indicates that the factor is not included in the operation. NEU#: Absolute neutrophil count, LYMPH#: Absolute lymphocyte count, MONO#: Absolute monocyte count, ESO#: Absolute eosinophil count, RDW: Red blood cell distribution width, PLT: Platelet, PDW: Platelet distribution width, PT: Prothrombin time, PTA: Prothrombin activity, PTINR: International Normalized Ratio, FIB: Fibrinogen, D-DIMER: D-dimer, HCY: Homocysteine, ALB: Albumin, PAB: Proalbumin, TG: Triglyceride, APOA/APOB ratio: The ratio of APOA to APOB, Lpa: Lipoprotein a. “OR”: Odds ratio. “C.I.”: Confidence interval. (DOCX) [file pone.0275350.s004.docx]

**S3 Table: Regression analysis of significant relative subitems to poor prognosis in AIS in step A, B, C.**

| Subitems | *P value^A^* | OR^A^ value (95% C.I. for OR^A^) | Subitems | *P value^B^* | OR^B^ value (95% C.I. for adjusted OR^B^) |
| --- | --- | --- | --- | --- | --- |
| NEU# (quintile) | <0.001* | 1.99 (1.68-2.35) | NEU# (quintile) | <0.001* | 1.71 (1.36-2.14) |
| LYMPH# (quintile) | <0.001* | 0.61 (0.53-0.71) | LYMPH# (quintile) | <0.001* | 0.64 (0.52-0.79) |
| MONO# (quintile) | <0.001* | 1.36 (1.19-1.56) | MONO# (quintile) | 0.01* | 1.30 (1.08-1.58) |
| ESO# (quintile) | <0.001* | 0.76 (0.67-0.88) | Age (quintile) | <0.001* | 1.32 (1.09-1.61) |
| RDW (quintile) | <0.001* | 1.33 (1.16-1.52) | D-dimer (quintile) | <0.001* | 1.94 (1.51-2.48) |
| PLT (quintile) | 0.05 | 0.88 (0.77-1.00) | **Subitems** | ***P value^C^*** | **OR^C^ value (95% C.I. for adjusted OR^C^)** |
| PDW (quintile) | 0.03* | 1.16 (1.01-1.33) | TIA | 0.04* | 0.12 (0.02-0.90) |
| PT (quintile) | <0.001* | 1.45 (1.26-1.67) | Cardiac disease | <0.001* | 2.45 (1.64-3.67) |
| PTA (quintile) | <0.001* | 0.60 (0.52-0.70) | Pneumonia | <0.001* | 4.81 (3.21-7.21) |
| PTINR (quintile) | <0.001* | 1.64 (1.42-1.90) | Babinski sign + | 0.01* | 1.67 (1.13-2.46) |
| FIB (quintile) | <0.001* | 1.22 (1.07-1.39) |  |  |  |
| HCY (quintile) | <0.001* | 1.37 (1.18-1.59) |  |  |  |
| ALB (quintile) | 0.04* | 0.85 (0.74-0.99) |  |  |  |
| PAB (quintile) | <0.001* | 0.69 (0.59-0.82) |  |  |  |
| TG (quintile) | 0.01* | 0.83 (0.72-0.95) |  |  |  |
| APOA/APOB (quintile) | 0.01* | 0.84 (0.72-0.96) |  |  |  |
| Lpa (quintile) | <0.001* | 1.49 (1.28-1.73) |  |  |  |
| Age (quintile) | <0.001* | 1.47 (1.27-1.68) |  |  |  |
| D-dimer (quintile) | <0.001* | 2.28 (1.90-2.73) |  |  |  |
| TIA | 0.01* | 0.09 (0.01-0.61) |  |  |  |
| Cardiac disease | <0.001* | 3.26 (2.23-4.77) |  |  |  |
| Pneumonia | <0.001* | 6.36 (4.31-9.38) |  |  |  |
| Hyperlipidermia | 0.02* | 0.51 (0.29-0.90) |  |  |  |
| Hyperhomocysteinemia | 0.03* | 1.59 (1.04-2.41) |  |  |  |
| Aphasia | <0.001* | 2.49 (1.44-4.31) |  |  |  |
| Babinski sign + | <0.001* | 1.98 (1.36-2.87) |  |  |  |

Note: A: Enter method to check single item. B: Only significant laboratory items were included for forward likelihood ratio detection. C: Only clinically significant items were included for forward likelihood ratio test. “/” indicates that the factor is not included in the operation. NEU#: Absolute neutrophil count, LYMPH#: Absolute lymphocyte count, MONO#: Absolute monocyte count, ESO#: Absolute eosinophil count, RDW: Red blood cell distribution width, PLT: Platelet, PDW: Platelet distribution width, PT: Prothrombin time, PTA: Prothrombin activity, PTINR: International Normalized Ratio, FIB: Fibrinogen, D-DIMER: D-dimer, HCY: Homocysteine, ALB: Albumin, PAB: Proalbumin, TG: Triglyceride, APOA/APOB ratio: The ratio of APOA to APOB, Lpa: Lipoprotein a. “OR”: odds ratio. “C.I.”: confidence interval.
