# Supplementary material for: Neutrophil count multiplied by D-dimer combined with pneumonia may better predict short-term outcomes in patients with acute ischemic stroke
Source: PLoS One. 2022 Oct 7;17(10):e0275350. doi: 10.1371/journal.pone.0275350 (PMC9543623; doi:10.1371/journal.pone.0275350)
Supplement: Attachment — Submitted filename: PONE-D-22-07312.docx [file pone.0275350.s006.docx]

***The product of neutrophils and D-dimer combined with post-stroke pneumonia may better predict short-term outcomes in patients with acute ischemic stroke***

**General comments:**

In this cross-sectional analysis, Xing et al. compared the clinical and laboratory test profiles of acute ischemic stroke patients who experienced “well” versus “poor” short-term (1 month follow-up) outcomes, defined as poor state, drowsiness, lethargy, coma, or death. The authors report that among evaluated factors, the product of neutrophil and D-dimer levels as well as post-stroke pneumonia showed the strongest association with poor outcomes. The inclusion of a comprehensive panel of clinical conditions and laboratory tests are strengths of the study. In addition, the evaluation of multiple laboratory sub-items as well as derived ratios and products is systematic and provides a level of detail that is not included in all similarly focused studies. The authors also provide a thorough discussion of the potential biological underpinnings for their work and findings, which is advantageous for readers whose background is not in this domain.

In future iterations of this work, a more rigorous description of the study design and methodology is highly recommended, particularly in regards to how the data was abstracted for analysis and how the outcome and other variables were defined. In its current form, readers may have difficulty following along with the study’s design or potentially trying to reproduce the findings. Although the study of clinical and lab-based biomarkers is highly relevant to current research on predicting stroke outcomes, the value that the currently reported findings adds to the literature may be limited, due to its narrow study setting and design. The authors should clearly articulate the strengths of the current study’s findings in comparison to prior studies that have also evaluated the predictive utility of lab-based biomarkers (e.g., D-dimer levels alone, neutrophil-to-lymphocyte ratio [NLR], platelet-to-lymphocyte ratio [PLR]). There are minor grammatical / syntactical errors / incomplete phrases throughout the manuscript. It is suggested that the authors review to improve readability.

Specific comments for each section of the manuscript are provided below.

**Introduction:**

- The introduction can be kept brief and concise, but the authors should more clearly articulate what is known or has been reported about the utility of clinical or lab-based biomarkers for predicting stroke outcomes, as this is the stated focus of the study. This would also help the reader understand what research gaps that the current study is seeking to fill.

**Methods:**

- Section 2.1: In addition to patient enrollment information, the authors should clearly describe the study design (e.g., prospective vs retrospective; cross-sectional) and define all outcomes and endpoints (e.g., primary, secondary), perhaps in a separate section of its own.
- Section 2.2.1: This section lists out the clinical information collected during the hospitalization. At the end of the section, the authors briefly mention “short-term poor outcomes” and in parentheses list out the following: “poor state, drowsiness, lethargy, coma, death, in 1 month.” If these are the criteria for poor outcomes used in the study, it would be advantageous to provide more detail on how these data are collected and characterized (e.g., via discharge diagnosis, clinical notes). Is a minimum of just 1 of these criteria needed to be flagged as having a poor outcome? To improve readability, consider including these details in a separate section where you define the main study outcomes.
- Section 2.2.2: Given the focus of the study on the predictive value of clinical and laboratory measures, the authors should clarify how the information was abstracted and transformed for analysis. Was the average of all values during the course of the hospitalization calculated? Include further details such that reproducibility of the study conduct is potentially easier.
- Section 2.2: Be very precise about the units measured for any clinical laboratory information. For example, in Section 2.2.3, reference values for D-dimer are given without units, and it is not clear if the detection units are the same.
- Section 2.3: Related to comment above. The authors mention comparisons between “Group P (poor outcomes) and Group W (well outcomes)” but should clearly describe how these groups were defined and / or what threshold was used. Was categorizing poor vs well an either / or situation (i.e., any patient who did not have a “poor outcome” was considered a “well outcome”)?
- Section 2.3: For the regression analyses, was there a primary independent or exposure variable considered and defined?

**Results:**

- Consider changing the heading for Section 3.2 or combine the findings with the previous section. It may be confusing to write that Section 3.2 is comparing the “performance of multiple laboratory items” when it is reporting descriptive differences.
- Section 3.3. The authors should describe the quintile analysis in more detail in the Methods. In Table 2, it is not clear which statistical difference that the P-value corresponds to. Is it referring to the difference in the continuous measurement data between Group P and Group W? Details for the quantitative results of the correlation test are not reported in the text. Overall, consider moving this table to the Supplement.
- Section 3.5. The authors make reference to “good regression relationship” between certain factors and poor AIS outcomes; however, the corresponding quantitative results that support this statement should be reported in the text.
- Section 3.4. In general, the text for this section is very dense as it includes results for both univariate and multivariate analyses. Although it is understood that the results of the univariate analyses inform the stepwise approach taken for the multivariate models, it is suggested that the section be split up. Making this change would also improve the readability of Table 3 and eliminate much of the “white space” that is currently present for the 3 multivariate models on the righthand side.
- Section 3.5. In Figure 4, consider ordering the legend from highest to lowest AUC; otherwise, the lines which are drawn in close proximity as well as the overall quality of the image may make it difficult for the reader to distinguish the findings.
- Section 3.6. Provide the criteria for defining post-stroke pneumonia in the Methods. This analysis is also not well-described and should be made more clear.
- In general, make sure all tables and table footnotes include appropriate detail for the reader to understand. Look over the figures and ensure all axes and legends are complete.

**Discussion / Conclusion:**

- The authors should include stronger discussion on how the study findings compare with current literature on the predictive value of lab-based biomarkers. For example, many studies have utilized D-dimer levels alone as well as derived values, such as neutrophil-to-lymphocyte ratio (NLR) or platelet-to-lymphocyte ratio (PLR). How does the neutrophil*D-dimer product (NDM) compare and can the authors provide an explanation for why it might serve as a better prognostic marker of poor outcomes?
- Consider additional limitations to mention. Although the focus of this study was on clinical and laboratory-based biomarkers, the authors should mention the lack of information on other known predictors of poor AIS outcomes (e.g., stroke severity).
- The authors suggest that their findings may differ from others that are conducted in “different latitudes”. Does this refer to patient populations in different geographies? Or are there other nuances in study design that this refers to? Please clarify.
- Include a brief comment on how the study findings can be implemented as part of the delivery of care.
